# *KIT* D816V Positive Acute Mast Cell Leukemia Associated with Normal Karyotype Acute Myeloid Leukemia

**DOI:** 10.1155/2018/3890361

**Published:** 2018-02-18

**Authors:** Marta Lopes, Maria dos Anjos Teixeira, Cláudia Casais, Vanessa Mesquita, Patrícia Seabra, Renata Cabral, José Palla-García, Catarina Lau, João Rodrigues, Maria Jara-Acevedo, Inês Freitas, Jose Ramón Vizcaíno, Jorge Coutinho, Luis Escribano, Alberto Orfao, Margarida Lima

**Affiliations:** ^1^Serviço de Hematologia Clínica, Hospital de Santo António (HSA), Centro Hospitalar do Porto (CHP), Porto, Portugal; ^2^Laboratório de Citometria, Serviço de Hematologia Clínica, Hospital de Santo António (HSA), Centro Hospitalar do Porto (CHP), Porto, Portugal; ^3^Unidade Multidisciplinar de Investigação Biomédicas, Instituto de Ciências Biomédicas Abel Salazar da Universidade do Porto (UMIB/ICBAS/UP), Porto, Portugal; ^4^Serviço de Anatomia Patológica, Hospital de Santo António (HSA), Centro Hospitalar do Porto (CHP), Porto, Portugal; ^5^Laboratório de Genética Molecular, Serviço de Hematologia Clínica, Centro Hospitalar do Porto (CHP), Porto, Portugal; ^6^Unidade de Biologia Molecular, Hospital de Santo António (HSA), Centro Hospitalar do Porto (CHP), Porto, Portugal; ^7^Sequencing Service (NUCLEUS), University of Salamanca, Salamanca, Spain; ^8^Institute of Biomedical Research of Salamanca (IBSAL), Salamanca, Spain; ^9^Biomedical Research Networking Centre Consortium (CIBER-CIBERONC), Madrid, Spain; ^10^Serviço de Hematologia Laboratorial, Hospital de Santo António (HSA), Centro Hospitalar do Porto (CHP), Porto, Portugal; ^11^Cytometry Service (NUCLEUS), University of Salamanca, Salamanca, Spain; ^12^Cancer Research Centre (IBMCC, USAL-CSIC), University of Salamanca, Salamanca, Spain; ^13^Department of Medicine, University of Salamanca, Salamanca, Spain

## Abstract

**Introduction:**

Mast cell (MC) leukemia (MCL) is extremely rare. We present a case of MCL diagnosed concomitantly with acute myeloblastic leukemia (AML).

**Case Report:**

A 41-year-old woman presented with asthenia, anorexia, fever, epigastralgia, and diarrhea. She had a maculopapular skin rash, hepatosplenomegaly, retroperitoneal adenopathies, pancytopenia, 6% blast cells (BC) and 20% MC in the peripheral blood, elevated lactate dehydrogenase, cholestasis, hypoalbuminemia, hypogammaglobulinemia, and increased serum tryptase (184 *μ*g/L). The bone marrow (BM) smears showed 24% myeloblasts, 17% promyelocytes, and 16% abnormal toluidine blue positive MC, and flow cytometry revealed 12% myeloid BC, 34% aberrant promyelocytes, a maturation blockage at the myeloblast/promyelocyte level, and 16% abnormal CD2−CD25+ MC. The BM karyotype was normal, and the *KIT* D816V mutation was positive in BM cells. The diagnosis of MCL associated with AML was assumed. The patient received corticosteroids, disodium cromoglycate, cladribine, idarubicin and cytosine arabinoside, high-dose cytosine arabinoside, and hematopoietic stem cell transplantation (HSCT). The outcome was favorable, with complete hematological remission two years after diagnosis and one year after HSCT.

**Conclusions:**

This case emphasizes the need of an exhaustive laboratory evaluation for the concomitant diagnosis of MCL and AML, and the therapeutic options.

## 1. Introduction

Mastocytoses are rare neoplasms defined by an abnormal expansion/accumulation of clonal mast cells (MC) in one or more organs or tissues [1, 2]. The 2016 revision to the “World Health Organization (WHO) Classification of Tumors of the Hematopoietic and Lymphoid Tissues” divides the disease into cutaneous mastocytosis (CM), systemic mastocytosis (SM), and localized mast cell tumors [1, 2]. Cutaneous mastocytosis includes maculopapular CM, also known as urticaria pigmentosa, diffuse CM, and mastocytoma of skin. Systemic mastocytosis is further divided into indolent SM (ISM), smoldering SM (SSM), and advanced SM variants; the latter includes aggressive SM (ASM), mast cell leukemia (MCL), and SM with associated hematological neoplasm (SM-AHN), previously known as SM with associated clonal hematological non-MC lineage disease (SM-AHNMD), and mast cell leukemia (MCL) [1, 2]. Associated hematological neoplasms may consist of myelodysplastic syndromes (MDS), myeloproliferative neoplasms (MPN), myelodysplastic/myeloproliferative neoplasms (MDS/MPN), and also acute myeloid leukemia (AML).

In the last years, serum tryptase levels [3], immunophenotypic characterization of BM mast cells by flow cytometry (FCM) [4, 5] and *KIT* mutation analysis [6], have proved to be useful for establishing the diagnosis, subclassifying and evaluating the prognosis of SM, adding important information to conventional cytomorphology and histopathology. Mast cells, and often other BM cells, from most patients with SM usually harbor the activating *KIT* D816V mutation. In addition, they regularly have abnormal morphology, from atypical MC type I, usually observed in ISM, to more immature atypical MC type II and metachromatic blasts, more frequently found in ASM and MCL, and they exhibit abnormal phenotypic features, from which the most frequent and more extensively studied are positivity for CD25 and/or CD2.

Mast cell leukemia accounts for <1% of all mastocytosis, it may appear de novo or secondary to a previously diagnosed MC disorder (usually ASM or SM-AHN), and it may associate with other hematological neoplasms (MCL-AHN) [7, 8]. Diagnosis is based on the presence of ≥20% atypical MC in the bone marrow (BM) and/or ≥10% in the peripheral blood (PB); an aleukemic variant with less than 10% of MC in the PB also exists. The European/American Consensus Group on Mastocytosis (EU/US-CGM) proposed a subclassification that distinguishes acute from chronic MCL based on the presence or absence of organ damage, respectively [9]. The neoplastic MC usually have an immature morphology and an abnormal immature and/or activated phenotype, although they often fail to have the abnormal CD2+CD25+ pattern encountered in most forms of SM; in addition, the *KIT* D816V mutation is detected in less than 50% of cases, and most patients have a normal karyotype. Symptoms of MC activation and involvement of the liver, spleen, peritoneum, digestive tract, and bones are relatively frequent, and skin lesions occur in only 1/3 of the cases. Treatment usually fails, and the median survival time is short. Due in part to the rarity, no standard therapy exists, and the role of hematopoietic stem cell transplantation (HSCT) needs further investigation.

Distinguishing MCL from SM associated with AML and from myelomastocytic leukemia (MML) is a challenge and requires a detailed laboratory investigation using cytology, cytochemistry, histopathology, immunohistochemistry, immunophenotypic, and genetic approaches [9–16]. The EU/US-CGM and the European Competence Network on Mastocytosis (ECNM) have recently proposed the criteria to establish the differential diagnosis between these entities [9].

We present a case of MCL diagnosed concomitantly to AML, given emphasis on the role of the laboratory exams, specially immunophenotyping and genetic testing, in establishing the differential diagnosis with MML. In addition, we compare the clinical and laboratory features of our patient with those described in previously published series of patients with MCL, ISM, ASM, and SM-ANH. Finally, we describe the success of the therapeutic strategy used in this patient, which included cladribine, anthracycline in combination with cytosine arabinoside, and HSCT.

## 2. Case Report

A 41-year-old Caucasian woman was admitted at the hospital with one-month history of asthenia, anorexia, fever, abdominal pain, early postprandial surfeit, and diarrhea. She had past history of an intermittent migratory pruritic maculopapular rash and mild episodes of flushing that had never been investigated, hypothyroidism, an anxiety disorder, and emotional instability, and she had smoked 20 cigarettes a day since the age of 13. There was no history of allergies or other pathologies.

When first observed at the hospital, she had a fever, a dark spot on the tongue, a slightly pruritic brownish erythematous maculopapular skin rash predominantly in the upper limbs, hepatomegaly (the left lobe of liver was enlarged and extended to epigastric region and the right lobe was four fingers below the right costal margin in the midclavicular line), and splenomegaly (5 fingers below the left costal margin). There was no peripheral lymphadenopathy.

**Figure 1:**
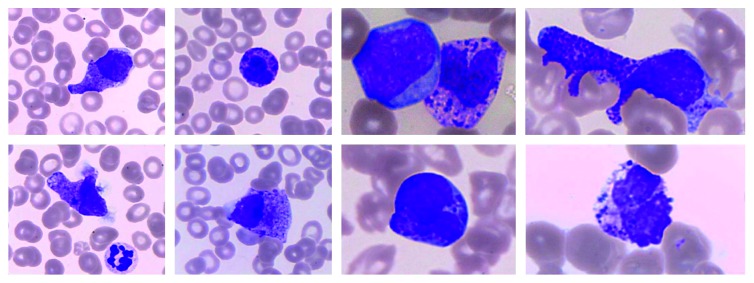
Peripheral blood smears showing different types of morphologically abnormal mast cells. Leishman's stain (a, 40x; b, 100x). Circulating mast cells (MC) had variable morphological features, from metachromatic blasts of medium to large cell size, round to oval shape, fine/immature nuclear chromatin, high nuclear : cytoplasmic ratio, and cytoplasmic metachromatic granules, to atypical MC type 2 with variable, sometimes spindle, cell shape, bilobated nucleus with fine to moderately condensed chromatin, variable nuclear : cytoplasmic ratio, and hypogranulated cytoplasm. Please note that in spite of these heterogeneous morphological features, all the MC present in the peripheral blood had an aberrant immature phenotype, corresponding to abnormal MC precursors (Figure 4).

Peripheral blood counts revealed pancytopenia: hemoglobin (Hg) 11.0 g/dl (normal range 12.0–15.0), platelets 16 × 10^9^/L (normal range 150–400), and white blood cells (WBC) 4.25 × 10^9^/L (normal range 4.0–11.0) with 7.0% neutrophils (0.3 × 10^9^/L) (normal range 2.0–7.5), 6% blast cells (BC), and 20% of cells with metachromatic cytoplasmic granules that were initially classified as basophils by morphology (Figure 1), but whose immunophenotypic study subsequently revealed them to be an abnormal immature MC.

Serum biochemistry showed elevated lactate dehydrogenase (369 U/L, normal range 135–214 U/L) and abnormal hepatic tests with a cholestatic pattern: total bilirubin 1.2 mg/dl (normal range: 0.2–1.0), direct bilirubin 1.1 mg/dl (normal range: 0.0–0.2), indirect bilirubin 1.14 mg/dl (normal range: 0.0–1.0), alanine transaminase 90 U/L (normal range: 10–30), aspartate transaminase 36 U/L (normal range: 10–30), alkaline phosphatase 731 U/L (normal range: 32–104), and gamma-glutamyl transferase 638 U/L (normal range: 6–39). There was also hypoalbuminemia (serum albumin 32 g/L, normal range: 35–50) and hypogammaglobulinemia (serum IgG 522 mg/dl, normal range: 793–1590; IgA 127 mg/dl, normal range: 114–457; IgM 170 mg/dl, normal range: 29–226). Serum tryptase levels were markedly increased (184 *μ*g/L, normal range < 13 *μ*g/L). Calcium and phosphate serum levels were normal, as did renal function tests. Coagulation tests, including prothrombin time, activated partial thromboplastin time, and fibrinogen levels, were within the normal range. Serological tests for hepatitis B and C viruses and human immunodeficiency virus type 1 and 2 were negative.

**Figure 2:**
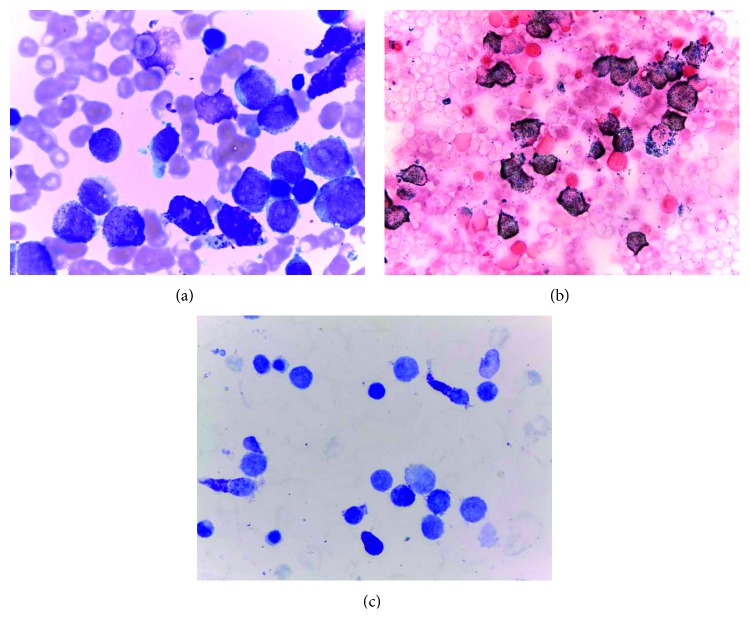
Bone marrow smears showing myeloperoxidase positive blast cells and promyelocytes, and morphologically abnormal toluidine blue positive mast cells. (a) Leishman. (b) Myeloperoxidase. (c) Toluidine blue staining.

**Figure 3:**
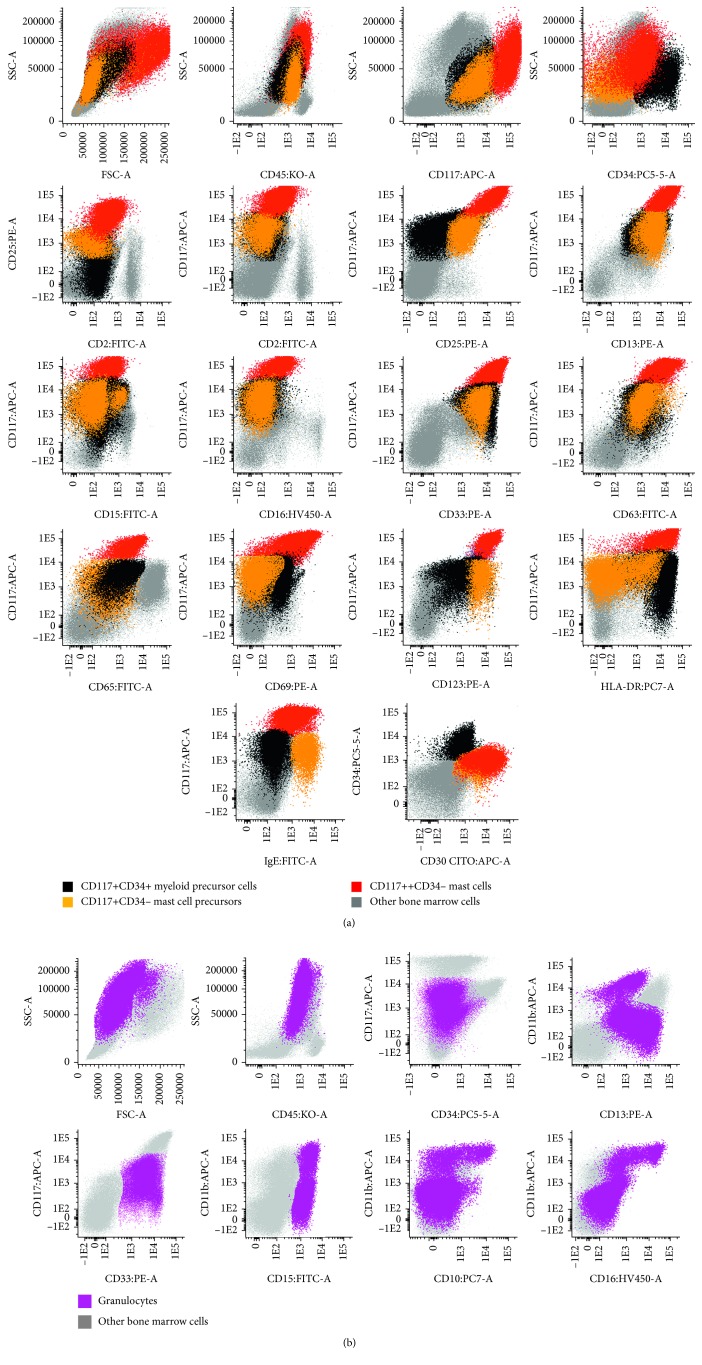
Bone marrow dot plots obtained by flow cytometry revealing 12% CD117+ CD34+ myeloid precursor cells (black dots), 5% CD117+CD34-mast cell precursors (orange dots), 13% of abnormal CD2− CD25+ mast cells (red dots), and 49% granulocytic cells with a maturation blockage at the promyelocyte level (purple dots). Myeloid precursor cells (black): CD117+, CD34+, CD2−, CD25−/+, CD13+, CD15−, CD16−, CD30−, CD33+, CD63+, CD65+low, CD69+low, CD123+, HLA-DR+high, and Fc*ε*RI/IgE−. Mast cell precursors (orange): CD117+, CD34−, CD2−, CD25+, CD13+, CD15−, CD16−, CD30+, CD33+, CD63+, CD65+low, CD69−, CD123+ high, HLA-DR−/+low, and Fc*ε*RI/IgE+. Mast cells (red): CD117+ high, CD34−, CD2−, CD25+ high, CD13+, CD15−, CD16−, CD30+, CD33+, CD63+, CD65+low, CD69+, CD123+ high, HLA-DR+high, and FcεRI/IgE+low.

**Figure 4:**
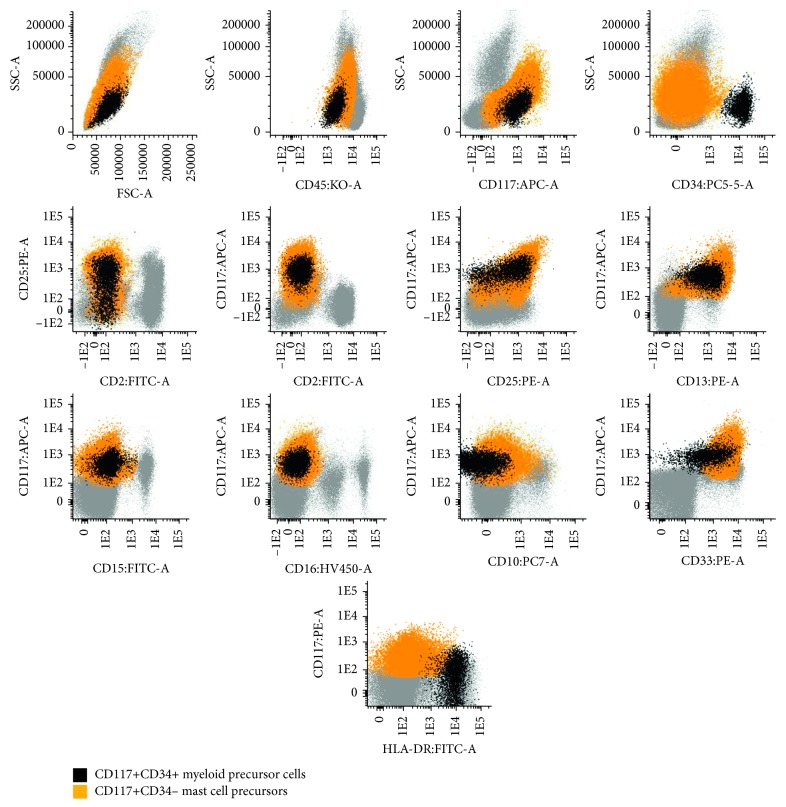
Peripheral blood dot plots obtained by flow cytometry showing 3% of CD117+ CD34+ myeloid precursor cells (black dots) and 49% of CD117+ CD34-mast cells precursors (orange dots). Please note that circulating myeloid precursor cells (black dots) and mast cells precursor (orange dots) had an immunophenotype like that observed in bone marrow (BM) myeloid precursor cells and BM mast cell precursors, respectively (please cf. Figure 3, same color code). Please also note that the relatively mature mast cells observed in BM (red dots, Figure 3) were not present in the peripheral blood.

Bone marrow smears showed 24% myeloperoxidase (MPO) positive BC, 17% promyelocytes, 4% myelocytes, 3% metamyelocytes + neutrophils (AML-M2 classification by cytomorphology), 30% erythroid lineage, and 16% morphologically abnormal toluidine blue positive MC (Figure 2). These cells had variable morphological features, from atypical MC type I and II to metachromatic blasts. There was no BM eosinophilia, or evidence of myelodysplasia. Flow cytometry of the BM aspirate revealed 12% of CD45+ (low), CD117+, CD34+ myeloid precursor cells (MPC) also expressing CD123, HLA-DR (high), CD13, CD33, CD65 (low), and CD25 (low, in part of the cells) but lacking CD10, CD15, CD16, CD2, CD30, and Fc*ε*RI/IgE; 5% of CD45+ (low), CD117+, CD34− MC precursors (MCP), also being positive for CD123 (high), CD13, CD33, CD65 (low), CD25, CD30, Fc*ε*RI/IgE, and HLA-DR (high), and lacking CD10, CD15, CD16, and CD2; 34% promyelocytes with an aberrant phenotype (CD45+, CD34−, CD117+, CD13+low, CD33+, CD65+; CD15+, MPO+, and CD2, CD10, CD11b, CD16, CD25, CD30, Fc*ε*RI/IgE, and HLA-DR negative); a maturation blockage at the promyelocyte level, as revealed by an abnormal CD11b/CD13/CD16 maturation pattern, with <1% of CD16+CD10+ mature neutrophils; and 13% of abnormal CD45+, CD34−, CD117+high MC with a relatively immature (CD123+high, Fc*ε*RI/IgE+ low, and HLA-DR+high), activated (CD63+, CD69+), and aberrant (CD2−, CD25+, and CD30+) immunophenotype (Figure 3). Cytoplasmic carboxypeptidase and surface CD203 were also positive (data not shown). In addition, FCM performed in PB, showed 3% CD45+low, CD117+, CD34+ MPC, and 21% of CD45+low, CD117+, CD34− MCP, which were phenotypically similar to the correspondent BM cell populations, at least for the cell surface markers tested, but not CD45+, CD34−, and CD117+ high MC (Figure 4).

**Figure 5:**
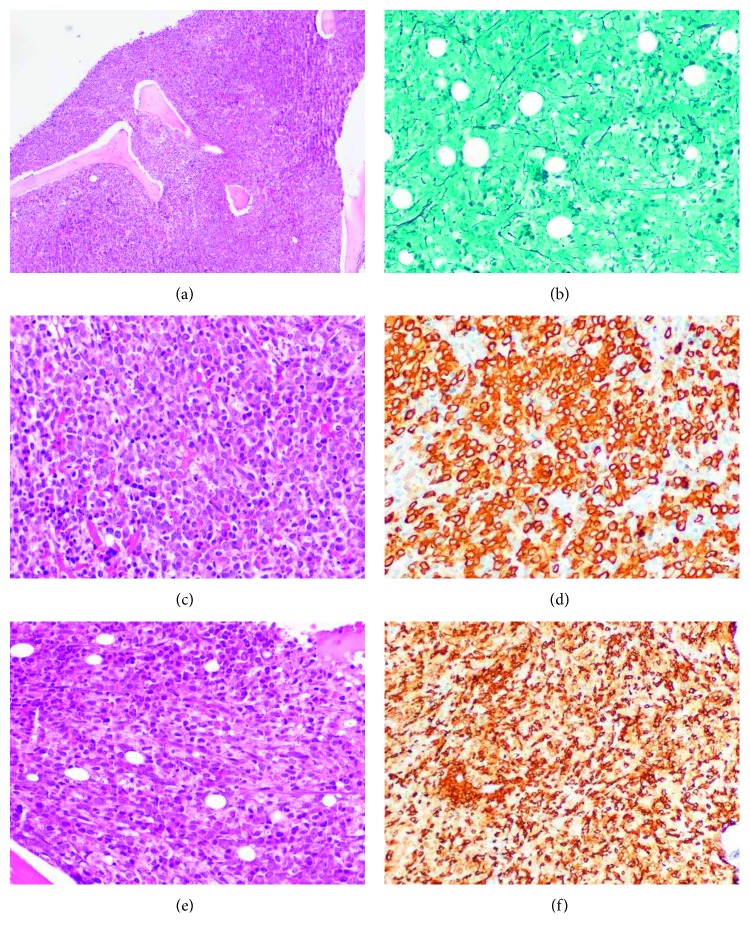
Bone marrow trephine biopsy showing a hypercellular marrow (a) with grade 2 fibrosis (b), and a preponderance of myeloid precursors with a diffuse proliferation of myeloperoxidase+ blast cells (c and d) and CD117+ mast cells (e and f). (a) Hematoxylin and eosin (H&E) stain, original amplification ×40. (b) Reticulin stain, original magnification ×100. (c) H&E stain, original amplification ×100. (d) MPO stain, original amplification ×100. (e) H&E stain, original amplification ×100. (f) CD117 stain, original amplification ×100.

Bone marrow trephine biopsy revealed a hypercellular marrow with increased proportions of immature MPO+ myeloid cells and morphologically atypical CD117+ fusiform MC forming perivascular dense aggregates, and grade 2 fibrosis (Figure 5). Skin biopsy was not performed.

Cytogenetic analyses of at least 20 Giemsa-banded BM cell metaphases obtained from unstimulated 24 hour cultures disclosed a 46,XX karyotype, without numerical or structural abnormalities. Genetic studies using probes for relevant targets, including t(15;17) PML-RARA, t(8;21) RUNX1-RUNX1T1, inv(16) CBFB-MYH11, and t(9,22) BCR-ABL, gave negative results. Tests for *FLT3* (FMS-like tyrosine kinase 3) and *NPM-1* (Nucleophosmin-1) gene mutations were also negative. *KIT* mutation at the codon 816 (D816V) (A7176T) was detected in all sorted BM cell populations, except in T cells; BM cells harboring the *KIT* D816V mutation included MC, CD34+ cells, CD34-HLA-DR-, CD34-HLA-DR+, and CD34-HLA-DR++ cells.

Abdominopelvic computerized tomography scan affirmed hepatomegaly (18.5 cm) and mild splenomegaly (13 cm) with small hypodense nodules (maximum diameter 10 mm) and revealed retroperitoneal adenopathies forming a conglomerate extending from the lesser gastric curvature and involving the large vessels; the largest adenopathy was in the hepatic-duodenal ligament and had 2.4 cm of major diameter. There was also a lamina of peritoneal liquid in pelvic cavitation.

Digestive endoscopy revealed slight reduced distensibility of the gastric body, which had a congestive mucosa with foci of erythema, and the duodenum had a congestive and micronodular mucosa. Biopsies were not performed due to severe thrombocytopenia. Skeleton radiography did not reveal osteolytic lesions. Thorax radiography had no evidence of mediastinal enlargement, lung consolidations, or pleural effusions.

**Figure 6:**
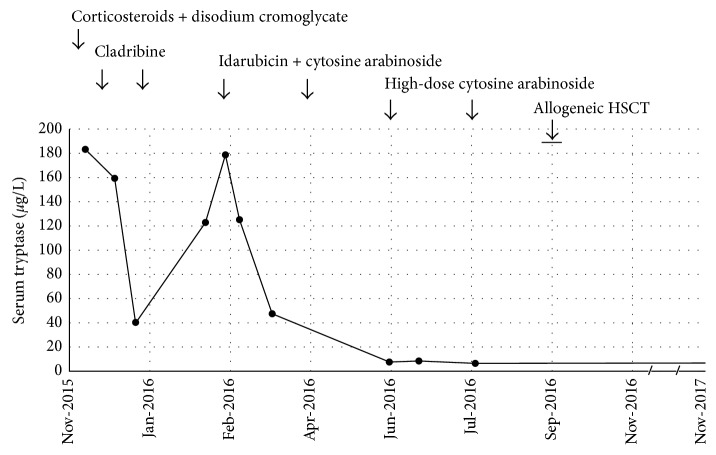
Serum tryptase levels from diagnosis of MCL-AML to date. Serum tryptase levels were markedly increased at the diagnosis (184 *μ*g/L, October 2015) and decreased transiently to 41 *μ*g/L after two cycles of cladribine (5 days, with one month of interval: December 2015; January 2016). However, one month later (February 2016), the serum tryptase levels increased again to 123 *μ*g/L, and the BM aspirate showed 47% myeloblasts. At that time, the patient received two cycles of combined therapy with idarubicin and cytosine arabinoside (3 + 7 days) (February 2016; April 2016) followed by two cycles of high-dose cytosine arabinoside (June 2016; July 2016). At day  +32 of reinduction chemotherapy (May 2016), the patient achieved complete hematological remission, and the serum tryptase levels decreased to 8 mg/L, remaining normal to date. At the end of August 2016, the patient received allogeneic hematopoietic stem cell transplantation (HSCT) from her sister. By the time of this report (November 2017, 24 months after the diagnosis, 15 months after HSCT), she maintains a complete hematological remission and a complete chimerism in peripheral blood.

According to the WHO criteria [1, 2], and to the consensus recommendations of the EU/US-CGM and the ECNM [9], the patient was diagnosed with *KIT* D816V+ MCL associated with AML with normal karyotype. She was immediately started with oral corticosteroids (prednisolone, 60 mg/day for one week, tapered to 20 mg/day over 1 month, and then maintaining 20 mg/day) and disodium cromoglycate (200 mg capsules, 4 times daily), and H1 (cetirizine, 10 mg/day, orally) and H2 (ranitidine, 150 mg twice a day, orally) antihistamines, which ameliorate the symptomatology. Then, she received two cycles of cladribine (0.14 mg/kg/day, administered over a 2-hour infusion for 5 days) with one month of interval, and the serum tryptase levels transiently decreased to 41 *μ*g/L (Figure 6).

One month after, she maintained constitutional symptoms, hepatomegaly, and pancytopenia, and she developed cutaneous and mucosal hemorrhage (petechial rash, epistaxis, and spontaneous oral cavity bleeding), myalgia, and bone pain. By that time, the serum tryptase serum levels had increased to 123 *μ*g/L (Figure 6), and the BM aspirate showed 47.0% myeloblasts, 8% promyelocytes, and 7.0% MC. Bone marrow FCM revealed 3% MPC (CD45+low, CD34+, CD117+, Fc*ε*RI/IgE−, CD2−, and CD25−/+), 7% MCP (CD45+ low, CD34−, CD117+, Fc*ε*RI/IgE+low, CD2−, and CD25+), 46% of immature granulocytic cells (almost complete maturational arrest at the promyelocyte stage), and 7% of CD45+, CD34−, CD117+ high, CD2−, and CD25+ MC. Peripheral blood counts were WBC 2.07 × 10^9^/L, neutrophils 4.0% (0.08 × 10^9^/L), MC 41.0%, BC 9.0%; Hg 8.8 g/dl; and platelets 28 × 10^9^/L. Flow cytometry studies performed in the PB showed 48% CD45+ low, CD117+, CD34−, Fc*ε*RI/IgE+low, CD25+, CD2− MCP, 4% CD45+low, CD34+, CD117+, Fc*ε*RI/IgE−, CD25−/+, CD2− MPC; once again, circulating CD45+, CD34−, CD117+high, CD2−, CD25+ MC were not observed. By that time, she received induction therapy for AML consisting of two cycles of idarubicin (12 mg/m2/day, intravenous, for 3 days) and cytosine arabinoside (AraC) (100 mg/m2/day, intravenous, for 7 days), achieving hematological remission and normal tryptase levels after the second induction course (Figure 6). At that time, the BM smears were slightly hypocellular with 1.3% of BC and no MC. Bone marrow FCM studies detected 1.5% of CD117+ CD34+ MPC, 54% maturing granulocytic cells, from which 26% were promyelocytes, 53% were metamyelocytes and myelocytes, and 21% were mature neutrophils, and 0.03% were phenotypically abnormal MC (0.02% CD117+ CD34− CD2− CD25−/+low, Fc*ε*RI/IgE+ MCP, and 0.01% CD117+high CD34−, CD2−, CD25+, Fc*ε*RI/IgE+low MC). Consolidation therapy performed in the subsequent 2 months consisted of two courses of high-dose AraC (2 g/m2, intravenous).

As complication of treatment she had bartholinite, treated with piperacillin plus tazobactam, and metronidazole; oral mucositis grade II controlled with tramadol; febrile neutropenia with bacteremia by *Escherichia Coli* treated with piperacillin plus tazobactam; pneumonia without respiratory insufficiency, which was responsive to imipenem plus vancomycin, and pseudomembranous colitis by *Clostridium difficile*, treated with metronidazole.

Two months after the second course of consolidation chemotherapy, the patient received isogroup HLA-identical related allogeneic HSCT from her sister (10/10 match) (5.09 × 10^6^/kg nonmanipulated peripheral blood CD34+ cells, totalizing 322 × 10^6^ CD34+ cells). The reduced-intensity conditioning regimen included fludarabine (30 mg/m2/day for 5 days) and busulfan (4 mg/kg/day for 2 days). As acute complication, she had febrile neutropenia treated with meropenem. On day 30 after HSCT, she had recovery of the hematological counts, and no myeloblasts or MC were seen in the PB. Unfortunately, the BM aspirate was hypocellular and results from BM studies were unevaluable. Abdominal echography revealed stable hepatomegaly (17.5 cm), without splenomegaly, or adenomegalies. Three months after HSCT a complete chimerism was documented in PB and BM neutrophils, monocytes, and lymphocytes. She developed a chronic graft versus host disease with cutaneous manifestations, controlled with cyclosporine A and mycophenolate mofetil. By the time of this report (24 months after the diagnosis, 15 months after HSCT), she maintains normal serum tryptase levels, complete hematological remission, and complete chimerism in PB (Figure 6).

## 3. Discussion

According to the WHO classification, this complex case fulfils the criteria for the diagnosis of SM-AHN, more precisely, *KIT* D816V+ MCL associated with normal karyotype AML [1, 2]. Criteria for MCL include not only the conditions for SM, such as BM infiltration by morphologically and phenotypically abnormal MC forming dense aggregates (>15 MC), increased serum tryptase levels (>20 ng/ml), and the *KIT* (D816V) mutation in BM cells, but also 16% MC in the BM and 20% MC in the PB; criteria for AML were more than 20% MPO+ myeloblasts in the BM by cytomorphology. Curiously, FCM studies showed that the cells of the MC lineage were phenotypically heterogeneous, with both aberrant MCP and more mature MC being identified in the BM, and only the former being present in the PB. Interestingly, FCM studies also revealed that the granulocytic cells had an almost complete blockage at the promyelocyte stage, despite the fact that t(15;17) was negative. Thus, the MC and the granulocytic cell lineages were both compromised by the leukemic process. In accordance to this multilineage involvement, the *KIT* D816V mutation was found in all BM cell populations tested, except in T cells. These findings are in line with previous studies, indicating that the occurrence of *KIT* mutations in an early progenitor cell results multilineage involvement, MC maturation blockade, immature MC phenotype, and aggressive disease [17–19]. The possibility of a secondary MCL arising in the context of a previously undiagnosed ISM is plausible, as the patient had a chronic maculopapular rash that had never been investigated. Unfortunately, skin biopsies were not performed, and thus, cutaneous infiltration by MC was not formally documented. It should however be mentioned, that secondary MCL usually occurs in patients with SM-AHN or ASM, and direct evolution from ISM to MCL is exceptionally rare [20–22].

The clinical and laboratory findings in patients with systemic MC neoplasms are diverse, depending on the disease subset and on the individual variability, and they are related to the release of MC mediators and/or to the infiltration of organs and tissues by the abnormal MC, which ultimately result in organ damage/failure (C-symptoms) [8, 20–22]. Except for a lower time from symptoms to diagnosis and a more severe neutropenia and thrombocytopenia, the clinical features observed in our patient with MCL + AML did not differ substantially from those usually found in patients with other advanced MC neoplasms, such as ASM and SM-AHN, as described in the largest series of patients with SM (342 cases), published by Lim et al. in 2009 (Table 1) [20]. In this series, ISM was the predominant SM subtype (46%), followed by SM-AHN (40%, subtype not specified) and ASM (12%), and only 4 cases were MCL (1%).

The clinical and laboratory features of our patient were those expected to occur in cases of MCL, as previously described in the literature (Table 2). In 2013, Georgin-Lavialle et al. revised all the MCL cases that had been published in scientific journals indexed in the MedLine [8]. In total, they provided data from 51 cases, including a series of 10 cases of MCL, published by Valentini et al. in 2008 [21], several cases reported individually from 1950 to 2012, and 4 personal unpublished cases; in 41 cases, they had enough data to classify them as de novo MCL (*n* = 30) or as secondary MCL (*n* = 11), and to compare the clinical and biological features of these entities (Table 2) [8]. Very recently, in 2017, Jawhar et al. reported on the clinical and laboratorial characteristics of 28 patients with MCL, from which 12 (43%) had secondary MCL and 20 (71%) had associated hematological neoplasms (MCL-AHN), other than AML [22] (Table 2). To the best of our knowledge, data available in the literature are not enough to establish if MCL-AHN has worse outcome than SM- (other than MCL) AHN, or if the type of AHN is more important for prognosis.

Rare cases of MCL have been diagnosed previously, subsequently or concomitantly to AML, as occurring in this patient [23–25]. Mast cell leukemia-AML cases exhibit a substantial increase (>20%) in myeloblasts in the BM, and they must be distinguished from MML where an AML may also be diagnosed, but criteria for SM are not met (Table 3).

Myelomastocytic leukemia is a very rare type of leukemia that is not yet incorporated in the WHO classification of the tumors of lymphoid and hematopoietic tissues [9, 16]. It is characterized by an expansion (>10%) of atypical MC together with BC with metachromatic granules (identified as MC precursors by immunophenotyping) in the BM and/or PB, concomitantly with criteria for an advanced myeloid neoplasm, which may have features of AML, MDS with excess of blast cells, or accelerated/blast phase of a MPN or a MDS/MPN. By definition, MML carries no specific (recurrent) molecular and immunophenotypic markers for SM. Specifically, activating point mutations at codon 816 of *KIT* are not found, and the aberrant CD2+/CD25+ MC immunophenotype, which is typically found in the majority of SM cases, is rare in MML. In addition, in patients with MML, the karyotype usually reflects the underlying disease (e.g., MDS, MPN, MDS/MPN, and AML) and chromosomal aberrations are frequently complex; in contrast, no recurrent chromosome abnormalities are known for patients with MCL [9, 16]. In addition, the neoplastic MC in MCL usually express CD117, low tryptase, and low Fc*ε*RI, and are often CD25+, whereas in MML, MC also express CD117 and tryptase, but they habitually stain negative for CD25. CD2 is usually negative in both cases, being frequently detected in MC from patients with indolent or smoldering SM (Table 4). Some other markers, including HLA-DR, CD30, and CD123, may also be positive in MCL cells, as observed in our patient. Thus, the clinical and laboratory findings observed in this case would favor the diagnosis of MCL associated to AML, instead of MML.

Management of patients with AML relies on genetic tests that allows for the diagnosis, informs about prognosis, and predicts response to therapy, and the value of genetics is reinforced in the WHO classification scheme for AML. For instance, cytogenetic aberrations have long been recognized as important prognostic variables in AML patients [26]. However, patients with AML and normal karyotype have had a very heterogeneous outcome, and previous studies have indicated that many other molecular aberrations do influence the response to treatment as well as in the risk of relapse [27]. For example, AML with normal cytogenetics may carry poor prognostic genetic lesions, such as *FLT3* mutations, overexpression of *BAALC* (brain and acute leukemia cytoplasmic), *ERG* (ETS/E26 transformation-specific-related gene), or *MN1* (meningioma 1) genes, or they may have aberrations that predict better prognosis as are cases with isolated *NPM-1* or *CEBPA* (CCAAT/enhancer binding protein alpha) mutations [27]. Among them, *FLT3* and *NPM-1* mutations were found to be absent in this patient. Also negative were studies for other relevant targets including t(15;17) PML-RARA, t(8;21) RUNX1-RUNX1T1, inv(16) CBFB-MYH11, and t(9,22) BCR-ABL. As mentioned before, *SRSF2*, *ASXL1*, and *RUNX1* mutations, described to be present in around 50% of MCL patients and found to affect adversely response to treatment and to predict lower OS [22], were not examined in this case.

Due fundamentally to the extreme rarity of MCL, no standard treatment exists, and therapies frequently consist on those commonly used in aggressive SM, such as cladribine (2-chloro-deoxy-adenosine, 2-CDA), interferon alpha 2a (IFN-*α*2a), and tyrosine kinase (TK) inhibitors (TKI). As MCL is presumably a clonal disorder of hematopoietic myeloid stem cells, chemotherapy with drugs proven successful in AML, such as anthracyclines in combination with AraC, have also been used to treat patients with MCL. If hematological remission is achieved, additional therapy with curative intent involving HSCT might be attempted, as in this patient [7, 8].

The rationale behind the use of cladribine, which was the first therapy tried in our patient, is based on the value of this drug in the treatment of aggressive SM, which has been confirmed in several studies [28, 29]. However, 2-CDA had no or little activity in this case, as in most previously reported cases of MCL treated with this purine analogue [22, 30], although in rare cases transient or prolonged partial response has been observed [21, 31, 32]. Reports on the use of IFN-*α*2a in MCL are scarce, and the results obtained were also not encouraging [21, 31].

Tyrosine kinase inhibitors have been actively investigated for the treatment of patients with mastocytosis because *KIT* mutations often cause constitutive activation of TK activity of the *KIT* receptor [33]. Imatinib is effective in patients with increased mast cells and eosinophils associated with *FIP1L1/PDGFRA* (Factor Interacting with PAPOLA and CPSF/Platelet-derived growth factor receptor A) fusion gene (e.g., myeloid neoplasm with eosinophilia and rearrangement of PDGFRA) [34] or rare patients with SM associated with *KIT* mutations outside of exon 17 [30]. However, the results from imatinib and other TKI, such as masitinib and dasatinib, in MCL and other D816V+ SM have been disappointing [21, 35–37].

Midostaurin (PKC412), a multitarget TKI, is a promising agent for patients with advanced SM, as it inhibits the growth of neoplastic MC exhibiting various mutant forms of *KIT*, including *KIT* D816V [38]. In contrast to other *KIT*-targeting drugs, midostaurin also impedes IgE-dependent release of histamine [39, 40]. Midostaurin has been reported to be efficacious in patients with advanced SM, including ASM and MCL [22, 41, 42]. Data from a Phase 2 single-arm open-label trial (CPKC412D2201), which included 89 with mastocytosis-related organ damage (16 with aggressive SM, 57 with SM-AHN, and 16 MCL), revealed that treatment with midostaurin 100 mg twice daily resulted in an overall response rate of 60% with a median duration of response of 24 months and a median overall survival of 29 months [42]. Unfortunately, midostaurin was not yet approved for patients with advanced SM, including MCL, and the drug was not available for compassionate use at the time the MCL was diagnosed in our patient. Brentuximab vedotin has also been considered for the treatment of patients with advanced SM. The results obtained in a small series of 4 patients with SM have suggested that this anti-CD30 monoclonal antibody-drug conjugate can induce durable responses with a manageable toxicity profile [43], and a clinical trial in patients with CD30 positive ASM and MCL is currently going on in the United States (NCT01807598). Thus, midostaurin and brentuximab vedotin may be considered alternative adjuvant therapies in case of disease relapse in our patient.

Induction chemotherapy has been used in some patients with MCL. According to the review performed by Georgin-Lavialle et al., AML-type chemotherapy was used in 6 of 51 MCL patients reported till 2013; the median survival time was 7 months, and all patients died between 2 and 29 months, because of disease progression or multiorgan failure [8]. A few cases of patients with MCL who received HSCT have also been reported, but sustained remission was not achieved in any of them [21, 44–46]. Ustun et al. have reported on a retrospective series of 57 patients with SM, including SM-AHN (*n* = 38), ASM (*n* = 7) and MCL (*n* = 12), who received allogeneic HSCT either after myeloablative (*n* = 36) or nonmyeloablative reduced-intensity (*n* = 21) conditioning regimens [47]. Responses were observed in 70% of the patients, with complete remission in 28%. Twenty-one percent of patients had stable disease, and 9% had primary refractory disease. The best responses were obtained in SM-AHN (AML/MDS), and the worst were obtained in MCL patients. Overall survival at 3 years was 57% for all patients, 74% for patients with SM-AHN, 43% for those with ASM, and 17% for those with MCL, and the strongest risk factor for poor overall survival was MCL. Survival was also lower in patients receiving nonmyeloablative compared with myeloablative conditioning regimens and in patients having progression compared with patients having stable disease or response. Although allogeneic HSCT may be considered a potentially curative treatment for advanced SM, including MCL, its definitive role needs to be determined by prospective clinical trials. Our patient maintains hematological remission of both diseases (MCL and AML) with complete chimerism, 15 months after HLA-identical HSCT.

In summary, we described an extremely rare case of an adult female with *KIT* D816V+ MCL associated with normal karyotype AML without *FLT3* and *NPM-1* mutations, who was refractive to cladribine and who achieved complete hematological remission after receiving induction chemotherapy with idarubicin and AraC, followed by allogeneic HLA-identical sibling HSCT preceded by a reduced-intensity conditioning regimen.

## Figures and Tables

**Table 1:** Clinical and laboratory features observed in our patient with MCL + AML, as compared to those described in a series of 342 patients with SM, including ISM, ASM, and SM-AHN, published by Lim et al. in 2009 [20].

	Lim et al. series [20]			This case (MCL-AML)
	ISM (*n* = 159)	ASM (*n* = 41)	SM-AHN (*n* = 138)	
*Demographic data*				
Age, years	49 (19–84)	65 (32–85)	65 (20–87)	41
Gender, males/females	69 (43)/90 (57)	19 (46)/22 (54)	97 (70)/41 (30)	Female
*Clinical features*				
Time from symptoms to diagnosis, months	72 (0–516)	18 (1–372)	15 (1–360)	1 month
Maculopapular skin lesions	100 (63)	15 (37)	25 (18)	Yes
Cutaneous symptoms	20 (71)	16 (62)	10 (83)	Yes
Constitutional symptoms	3 (19)	24 (59)	85 (62)	Yes
MC mediators-related symptoms	110 (69)	9 (22)	39 (28)	Yes
Anaphylactoid reactions	53 (33)	2 (5)	2 (1)	No
Musculoskeletal symptoms	48 (30)	17 (41)	41 (30)	No
Gastrointestinal symptoms	113 (71)	26 (63)	79 (57)	Yes
Hepatomegaly	22 (14)	16 (39)	53 (38)	Yes
Splenomegaly	26 (17)	18 (44)	76 (57)	Yes
Lymphadenopathy	22 (14)	11 (27)	40 (29)	Yes
*C-findings*	NA	41 (100)	36 (26)	Yes
BM dysfunction with cytopenia(s)	NA	13 (32)	NA	Yes
Hepatomegaly with functional impairment	NA	11 (27)	20 (14)	Yes
Splenomegaly with hypersplenism	NA	9 (22)	16 (12)	Yes
Osteolysis/pathological fractures	NA	18 (44)	5 (4)	No
Malabsorption with weight loss	NA	2 (5)	1 (1)	No
*Peripheral blood findings*				
Hemoglobin, g/dl	13.9 (8.1–16.7)	11.3 (5.1–16.5)	10.9 (6.4–17.4)	11.0
Hemoglobin, <10.0 g/dl	4 (3)	10 (24)	48 (45)	No
Platelets, ×10^9^/L	260 (39–570)	179 (20–561)	129 (2–1625)	16
Platelets, <100 × 10^9^/L	2 (1)	11 (27)	50 (37)	Yes
Neutrophils, ×10^9^/L	4.2 (0.6–12.4)	4.2 (0.9–17.8)	4.8 (0.2–42.5)	0.3
Neutrophils, <100 × 10^9^/L	2 (1)	2 (5)	11 (8)	Yes
Serum tryptase, *μ*g/L	53.6 (11.4–1410)	145 (10–2000)	75.4 (3.7–1360)	184
Increased tryptase: >11.5/>200 *μ*g/L	89 (99)/11 (12)	14 (93)/6 (40)	49 (92)/15 (28)	>11.5/<200
Decreased albumin (<35 g/L)	10 (9)	10 (26)	29 (27)	Yes
Increased AP (>115 U/L)	36 (25)	24 (60)	65 (50)	Yes
Increased AST (>48)/ALT (>55 U/L)	10 (7)/4 (7)	5 (13)/1 (9)	22 (17)/5 (16)	Yes
Increased total bilirubin	3 (11)	10 (28)	42 (32)	Yes
Increased LDH (>222 U/L)	2 (4)	1 (9)	25 (25)	Yes
*Bone marrow findings*				
BM cellularity: increased/decreased	49 (32)/15 (10)	24 (67)/3 (8)	123 (91)/1 (1)	Increased
MC in BM biopsy: <10%	58 (41)	9 (26)	42 (35)	NA
MC in BM biopsy: 10–30%/>30%	68 (48)/15 (11)	17 (50)/8 (24)	66 (55)/12 (10)	NA
Fibrosis grade 2 or more	5 (14)	8 (47)	31 (46)	Grade 2
MC phenotype, FCM: CD2+/CD25+	27 (66)/39 (95)	4 (50)/8 (100)	7 (33)/18 (86)	No/Yes
MC nuclear morphology: oval/elongated	87 (78)/24 (22)	21 (66)/7 (22)	82 (73)/17 (15)	Mixed
MC nuclear morphology: indented/round	0 (0)/0 (0)	3 (9)/1 (3)	10 (9)/3 (3)	
Blasts in BM smears: 5–10%/>10%	0 (0)/0 (0)	0 (0)/0 (0)	16 (12)/11 (8)	24%
*Molecular and chromosomal aberrancies*				
*KIT* D816V mutation	(78)	(82)	(60)	Yes
*FIP1L1-PDGFRA* rearrangement	(52)	NA	NA	No
*JAK2* V617F mutation	(4)	NA	NA	NA
Abnormal karyotype	(5)	(20)	(31)	No
*Survival and leukemic transformation*				
Transformation into AML or MCL	1 (<1)	2 (5)	18 (13)	MCL + AML
Median survival time from diagnosis, mo	198	41	24	Alive, 24 mo
Deaths after median follow-up of 21 mo	26 (16)	25 (61)	99 (72)	NA

AHN, associated hematological neoplasm; ALT, alanine transaminase; AML: acute myeloid leukemia; AP, alkaline phosphatase; AST, aspartate transaminase; BM: bone marrow; FCM, flow cytometry; LDH, lactate dehydrogenase; MC, mast cells; MCL, mast cell leukemia; mo, months; NA, not available, not evaluated or not applicable. Not all parameters were evaluated in all patients. Results are presented as median (range) values or as number (percentage) of patients with the mentioned characteristic.

**Table 2:** Clinical and laboratory features observed in our patient with MCL + AML, as compared to those described in a review of 51 cases of MCL performed by Georgin-Lavialle et al. in 2013 [8], and in a series of 28 patients with MCL published by Jawhar et al. in 2017 [22].

	Georgin-Lavialle et al. review [8]			Jawhar et al. series [22]			This case (MCL-AML)
	MCL (*n* = 51)	De novo MCL (*n* = 30)	Secondary MCL (*n* = 11)	MCL (*n* = 28)	De novo MCL (*n* = 16)	Secondary MCL (*n* = 12)	
*Demographic data*							
Age, years	52 (5–76)	52 (18–76)	35 (5–75)	67 (45–82)	69 (47–82)	65 (45–73)	41
Gender, males/females	20 (40)/30 (60)	11 (38)/18 (62)	6 (55)/5 (45)	16 (57)/12 (43)	10 (63)/6 (37)	6 (50)/6 (50)	Female
*Diagnosis*							
MCL (without AHN)	36/40 (90)	27 (89)	11 (100)	8 (29)	6 (38)	2 (17)	No
MCL-AHN (other than AML)^∗^	4/40 (10)	3 (11)	0 (0)	20 (71)	10 (62)	10 (83)	No
MC disorder prior to MCL^∗∗^	11 (40)	0 (0)	11 (100)	12 (43)	0 (0)	12 (100)	Probably yes
ASM	NA	NA	NA	2 (7)	0 (0)	2 (17)	No
SM-AHN	NA	NA	NA	10 (36)	0 (0)	10 (83)	No
Leukemic MCL (MC in the PB ≥ 10%)	18/47 (38)	15 (50)	3 (30)	2 (7)	NA	NA	Yes (20)
*Peripheral blood findings*							
Tryptase, *μ*g/L	433 (21–2357)	433 (21–742)	250 (173–2357)	520 (157–1854)	520 (157–1854)	544 (160–1250)	184 *μ*g/L
Tryptase > 200 *μ*g/L	NA	NA	NA	26 (93)	15 (94)	11 (92)	No
Cytopenias	NA	NA	NA	26 (93)	15 (94)	11 (92)	Yes
Hemoglobin, gr/dl	9.9 (5.4–14.0)	9.0 (5.4–13.7)	11.0 (8.1–13.3)	8.9 (7.9–14.3)	9.2 (7.9–13.3)	8.7 (7.9–14.3)	11.0
Neutrophils, ×10^9^/L	3.7 (1.0–15.3)	6.0 (1.0–14)	NA	NA	NA	NA	0.3
Platelets, ×10^9^/L	110 (5–318)	82 (5–202)	111 (30–150)	69 (21–795)	64 (21–795)	86 (26–331)	16
Hypoalbuminemia < 35 g/L	NA	NA	NA	11 (39)	5 (31)	6 (50)	Yes (32 g/L)
Alkaline phosphatase > 150 U/L	NA	NA	NA	20 (71)	10 (62)	10 (62)	Yes (731 U/L)
% Mast cells	6 (0–72)	7 (0–72)	0 (0–12)	NA	NA	NA	21%
*Other relevant findings*							
Splenomegaly	38/49 (65)	23 (82)	9 (82)	28 (100)	16 (100)	12 (100)	Yes
Hepatomegaly	32/47 (68)	21 (78)	6 (60)	NA	NA	NA	Yes
Skin lesions	15/50 (30)	4 (14)	5 (45)	NA	NA	NA	Yes
Ascites	9/50 (18)	5 (17)	1 (9)	13 (46)	7 (44)	6 (50)	Yes
Asthenia	NA (78)	NA	NA	NA	NA	NA	Yes
Weight loss > 10% in 6 months	NA (38)	NA	NA	12 (43)	8 (50)	4 (33)	NA
Anorexia	NA (20)	NA	NA	NA	NA	NA	Yes
Flushing and other MCAS	39/50 (78)	24 (83)	8 (73)	NA	NA	NA	Yes
*Bone marrow findings*							
% MC in BM smears	NA	NA	NA	25 (20–95)	25 (20–95)	20 (20–95)	16
% MC in BM biopsy	50 (20–100)	60 (20–100)	60 (25–90)	65 (20–95)	60 (20–95)	65 (30–95)	NA
*Phenotypic aberrancies*							
CD2+	15/29 (52)	6 (46)	1	NA	NA	NA	No
CD25+	18/24 (75)	6 (50)	3	NA	NA	NA	Yes
*Molecular and chromosomal aberrancies*							
*KIT* D816V mutation	13/28 (46)	6 (40)	1 (20)	16 (68)	10 (63)	9 (75)	Yes
Other *KIT* mutations	6/28 (22)	3 (19)	3 (60)	6 (21)	4 (25)	2 (17)	NA
*SRSF2*, *ASXL1*, or *RUNX1* mutations	NA	NA	NA	13 (52)	7 (50)	6 (55)	NA
Normal karyotype^∗∗∗^	20/23 (83)	8 (73)	NA	19 (79)	11 (79)	8 (10)	Normal
*Survival and leukemic transformation*							
Survival, months	6 (0.5–98)	4 (0.5–24)	5 (1–18)	17 (1–86)	NA	NA	>24
Progression into secondary AML^∗∗∗∗^	NA	NA	NA	3 (11)	2 (13)	1 (8)	MCL + AML
Deaths	33/48 (69)	24 (83)	5 (62)	18 (64)	10 (63)	8 (67)	Alive

AHN, associated hematological neoplasm; AML, acute myeloid leukemia; BM, bone marrow; CEL, chronic eosinophilic leukemia; CM, cytomorphology; CMML, chronic myelomonocytic leukemia; MC, mast cells; MCAS, mast cell activation-related symptoms; MCL, mast cell leukemia; MCL-AHN, mast cell leukemia with associated hematological neoplasm; MDS, myelodysplastic syndrome; MDS/MPN, myelodysplastic myeloproliferative neoplasm; NA, not available or not evaluated. Results are presented as median (range) values or as number (percentage) of patients with the mentioned characteristic. Not all parameters were evaluated in all patients. ^∗^AHN included MDS (*n* = 3) and CMML (*n* = 1) in the Georgin-Lavialle review, and CMML (*n* = 8), MDS/MPN unclassifiable (*n* = 5), MDS (*n* = 5) or CEL (*n* = 2) in the Jawhar series. ^∗∗^Our patient had past history of uninvestigated maculopapular skin lesions and flushing episodes, and a maculopapular rash at the diagnosis (skin biopsy not performed). ^∗∗∗^In the Jawhar series, karyotype was available in 24 patients with MCL (14 patients with de novo MCL and 10 patients with secondary MCL); 5 patients (21%) had an aberrant karyotype (3 patients with de novo MCL, 21%; 2 patients with secondary MCL, 20%), with 3 patients having a complex karyotype (≥3 aberrations) and 2 patients having del(5q) or del(12p), respectively. Cytogenetic studies were available for 23 of 51 cases reviewed by Georgin-Lavialle et al., and a normal karyotype was found in 83% cases (83%). Two patients (9%) had a 5q deletion and were diagnosed as having MCL-MDS, and 2 patients with de novo MCL had t(10;16)(q22;q13q22) and t(8;21)(q22;q22). Thus, there are no recurrent cytogenetic abnormalities in MCL. ^∗∗∗∗^Three patients from the Jawhar series progressed into secondary AML 18, 28, and 34 months, respectively, after the diagnosis of MCL had been established.

**Table 3:** Clinical and laboratory features in our patient as compared to those observed in patients with MCL or MML, as schematized by Valent et al. in 2014 [9].

	Mast cell leukemia (MCL)	Myelomastocytic leukemia (MML)	This case (MCL-AML)
*Clinical features*			
Skin lesions	Present in a subset of patients	Absent	Yes (maculopapular rash)
Spleen involvement/splenomegaly	Found in a subset of patients	Usually present at diagnosis	Yes (13 cm; splenic nodules)
Liver involvement/hepatomegaly, ascites	Often found	Usually not found	Yes (18.5 cm; cholestasis; ascites)
MC mediators-related symptoms	Frequent	Frequent	Yes (flushing; diarrhea)
*Peripheral blood findings*			
Serum tryptase (*μ*g/L)	Markedly elevated (usually >200; often >500)	Moderately elevated (usually <100; often <50)	184 *μ*g/L
Circulating MC	Present in a subset of patients	Present in a subset of patients	Yes (CM: 20%); FCM: 21% MCP
Circulating myeloblasts	No (except MCL-AHN)	Present in a subset of patients	Yes (CM: 6%); FCM: 3% MPC
*Bone marrow findings*			
Underlying non-MC myeloid neoplasms	No (except MCL-AHN)	Yes	AML (concomitant diagnosis)
Increased myeloblasts	No (except MCL-AHN)	Almost always seen	Yes (CM: 24% myeloblasts + 17% promyelocytes); FCM: 12% MPC + 34% promyelocytes
MC clusters and sheets in BM biopsy	Yes	No	Yes
Diffuse MC infiltrate in the BM biopsy	Yes	Yes	Yes
MC in BM smears	≥20%	≥10%	Yes (CM: 16%); FCM: 5% MCP + 13% MC
Karyotype	Normal or abnormal with a few lesions (except MCL-AHN)^∗∗^	Usually complex^∗∗^	Normal
CD25+ MC	Yes	No	Yes
*KIT* D816V or other codon 816 mutation	Present	Not found	Yes (BM mast cells, CD34+ precursors and other myeloid cells)
*KIT* mutations in non-816-codons	Found in a subset of patients	Found in a subset of patients	Not investigated

AHN, associated hematological neoplasm; AML, acute myeloid leukemia; BM, bone marrow; CM, cytomorphology; FCM, flow cytometry; MC, mast cells; MCL, mast cell leukemia; MCL-AHN, mast cell leukemia with associated hematological neoplasm; MCP, mast cell precursors; MML, myelomastocytic leukemia; MPC, myeloid precursor cells. ^∗^Past history of uninvestigated maculopapular skin lesions and flushing episodes; maculopapular rash at the diagnosis (skin biopsy not performed). ^∗∗^In MML, the karyotype usually reflects the nature of the underlying disease, whereas no recurrent chromosome abnormalities are known for patients with MCL.

**Table 4:** Cell molecules expressed in MC from our patient as compared to those usually expressed in the MC from patients with MCL or MML, as schematized by Valent et al. in 2014 [9].

Markers	Normal MC	ISM	SSM	ASM	MCL	MML	This case
CD34 (HPCA-1)	−	−	−	−	−	−	−
CD117 (*KIT*)	+	+	+	+	+	+	+
Tryptase	+	+	+	+	+	+	NA
CD33 (Siglec-3)	+	+	+	+	+	+	+
CD123 (IL-3RA)	−	−	−	+/−	+/−	−	+
CD2 (LFA-2)	−	+/−	+/−	−/+	−/+	−	−
CD25 (IL-2RA)	−	+	+	+	+	−	+
CD30 (Ki1)	−	−/+	+	+	+	NA	+
FcεRI	+	+	+	+	−/+ (low)	−	+ (low)

HPCA-1, human precursor cell antigen-1; IL-2RA, interleukin-2 receptor alpha chain; IL-3RA, interleukin-3 receptor alpha chain; ISM, indolent systemic mastocytosis; SSM, smoldering SM; ASM, aggressive SM; MCL, mast cell leukemia; MML, myelomastocytic leukemia; NA, not available. +, expressed in MCs in almost all (>90% of) cases; +/−, expressed in a majority of MC in a considerable proportion of cases; −/+, expressed in a minority of MC in a smaller subset of cases; −, not expressed. In a few cases, one of the two markers, either CD2 or CD25, may be expressed in neoplastic MCs in MML.
